# Opinions of professors, dental students, and patients for publishing the patient images in the articles

**DOI:** 10.1186/s12903-023-03792-4

**Published:** 2024-01-03

**Authors:** Ava Rowshani, Maryam Alsadat Hashemipour

**Affiliations:** 1 Social Determinants on Oral Health Research Center, Kerman University of Medical Sciences, Kerman, Iran; 2 Oral and Dental Diseases Research Center, Kerman University of Medical Sciences, Kerman, Iran

**Keywords:** Patient, Image, Dental student, Journal, Personal data

## Abstract

**Introduction:**

The journals must have an instruction for writers to observe the essential ethical principles like privacy-preserving, secrecy, and keeping the patients’ identities hidden. Even though patient secrecy is an important ideology in medicine’s ethics, most journals have a little guide on this topic for the authors. According to the absence of such studies in dentistry and limited studies in medicine, our goal in this article is to review the opinions of professors, Kerman dentistry students, and patients for publishing the patient images in the articles.

**Method:**

This research is an analytical, sectional, and descriptive study. The studied society includes the professors of the dentistry faculty (54 people), the 4th to 6th years dentistry students (122 people), and 129 patients who referred to the offices, the faculty, and other clinics in Kerman city base on simple random sampling method. A query including the personal questions, and questions related to the participants’ opinions about publishing the images was given to contributors. Abundance, average tables, chi-square (χ 2) test, T-test, and SPSS 21 software were used for data description.

**Results:**

The contributors’ attitudes were different in three groups of participants: more than half of the patients (58.91%), 39.5% of students, and 31.38% of professors believed that no permission is needed. While, 64.34% of the patients, 89.34% of students, and 83.3% of professors believed that written permission is needed for publishing.

**Conclusion:**

From the participants’ viewpoints, more strict forms are needed by increasing identity recognizability. The professors are more eager than the patients to receive patients’ permission for any kind of image. By reducing the level of identification, doctors and students are more eager than patients to receive approvals.

## Introduction

The journals that publish clinical studies or case reports may contain images of patients that sometimes cause the patient to be identified by others. Twenty years ago, science journals were only available in academic libraries, but nowadays, almost every literary journal is generally online, and many researchers and readers use open access. Unfortunately, open access will allow anybody to use a picture without permission. Although clinical doctors and patients increasingly use the internet to search for medical information, whether they know that reusing a clinical picture is not legal is unclear [1–3].

Although patients have high confidence in doctors, specialists are obligated to provide the best information to the patients, allowing them to choose wisely. This matter applies to choosing the treatment and participating in researching and publishing images of individuals in journals. Also, journals must have clear policies and guidance for authors in observance of ethical standards such as keeping privacy, being anonymous, and confidentiality of patients. Although patience and confidentiality are essential, many journals give little guidance to the authors in this matter or no direction at all [2, 3]. In many cases, there is no guidance in this matter [4, 5].

Research has shown that medical images of patients are a vital part of the patient’s medical reports and must be regarded with reliability considerations and individual privacy [6–8]. It is not always possible to deidentify patients’ images; therefore, they must not be published in journals without the patient’s Informed Consent [9–14].

Medical images’ primary usage (application) relates to patients’ diagnosis, treatment, and follow-up. In contrast, their usage for educational and research purposes is considered a second priority [7, 8]. Therefore, knowledgeable consent is an essential part of the research procedure and needs to be more than a signature and patients’ informed consent must be taken freely and without force and based on a clear understanding of the participants [15].

In some countries, for example, Britain and USA, there are federal and professional instructions for publishing images in academic resources and social networks [16–21]. Due to recent legalization in the EU, medical images and other personal data must be deidentified as quickly as possible for medical research purposes; this process means that without additional separate information, personal data cannot be related to an individual [12]. It is emphasized that individuals must be aware that after publishing images on the general platform, it is impossible to control further usage of the pictures. If consent is determined for a specific use, it cannot be generalized for other purposes. Some journals have policies that in addition to the permission of participate in research; separated patient consent is needed for publishing medical images [22].

Also, the face de-identifying image techniques are not standardized yet. X-rays or small partial images of the face or mouth have less potential for identification than cases with full face images. A traditional way to keep anonymity in the image of a patient’s face is the putting a black ribbon on the patient’s eye [4].

In some situations, identification is not possible; in these circumstances, the patient must be explained entirely to give consent to publishing images [23–25]. Special attention to trust and privacy is necessary to use the patient’s image. Even though policies and processes for publishing patient images already exist, there is little systematic evidence of patients’ and health specialists’ knowledge of the necessity of consent for publishing medical photos. The self-determination, privacy, and confidentiality of patients must be respected. Patient consent is necessary for any possible use in all cases where images and pictures contain identifiable information. The patients must be aware that when an image is published with evolving electronic publishment, efficient control of miss abuse will not exist in the future. Doctors and hospitals must use any part of the patient’s medical history as confidential.

They must try to use images and pictures being employed in the teaching anonymously to make no moral or legal concerns. For using images and photos in the journal, medical education must respect moral laws and contain anonymous information to prevent legal consequences. Therefore, due to the lack of studies on this matter in the dentistry field and limited studies in medical fields, this study is the first one in Iran and even in the Asia region. It is important that 3 groups of patients, students and specialist dentists who are somehow involved in the subject have been included in the study. The purpose of this research is to review the professor’s comments, Kerman dentistry students, and patients on the subject of publishing patient images in journals.

## Methods

According to the null hypothesis in this research, there was no any relationship between the attitudes of the professor, students, and patients on the subject of publishing patient images in journals. Besides, the alternative hypothesis was that there is a relationship between the attitudes of the professor, students, and patients on the subject of publishing patient images in journals.

This research is a cross-sectional, descriptive-analytical study based on the simple random sampling method. The studied communities were professors of dentistry college (45 cases), 4th to 6th-year students (122 cases), and 129 patients visiting medical offices, colleges, and clinics across the city of Kerman. About the sample size the value of related parameters are: z = 1.96, *p* = 0.07, q = 0.03, d = 0.07 where d is the allowed error value (error value), *p* equals to the estimated proportion of the trait in the community, or in other words, *p* the probability of the presence of that trait in the community and Q is equal to the improbability of the attribute, which is obtained by Q = 1-p. Finally Z is a constant number whose value is always 1.96.

First, the lists of students and professors were provided from the education department and the recruitment unit, respectively. Then, the checklist was completed by researchers based on previous studies. The entry criterion was being professors and students in the college, and the exit criterion was the lack of people interested in completing the questionnaire. Inclusion criterion was the willingness to fill out the questionnaire, dental student and dentist, while, the exclusion criterion was the reluctance to fill out the questionnaire. For setting the questionnaire validity, the questionnaire has been reviewed by 4 college professors, such that the validity and reliability were approved. The questionnaire was given to 20 students and patients over ten days, and Cronbach’s alpha factor was 0.81, which showed that the validity was suitable.

The questionnaire contained personal questions and questions related to participants’ opinions about publishing patient images by senior students. Verbal consent was taken from each participant and the purpose of the study was explained. If she/he was in agree for entering into study, the questionnaire was presented to the individuals. Also, everyone who was issued questionnaire information will remain confidential and be studied statistically. Written consents are preferable in cases including long-term follow-ups, high-risk interventions, and cosmetic procedures, and in questionnaire based studies, oral consent can be obtained from the individuals.

The data were described using the frequency table, average table, Chi-squared test, T-test, and SPSS 21 software. Chi-square was used to evaluate the response of 3 groups of participants in relation to need of total consent and need for oral or written consent and its relationship with demographic variables.

## Results

This research includes 305 respondents [129 patients (129 people out of 140 people, response rate = 92.1), 122 students (122 stuents out of 136 students, response rate = 89.7), and 54 professors(54 people out of 62 people, response rate = 87.1)]. The average age of patients was 52 years old, 23 years old for students, and 38 years for professors (the total average was 7.86 ± 37.92). In all three groups, the number of women was more than men. Most of the patients were high school graduates or had university degrees. Less than one-quarter of the professors (24.7%) had published an article, and less than 10.8% had the experience of reading an article with pictures. Table 1.

**Table 1:** Demographic characteristics of participating

Variable		Patients		Students		Teacher	
		No	%	No	%	No	%
Quantity		129	42.30	122	40	54	17.70
Gender	Female	74	24.26	81	26.56	34	11.15
	Male	55	18.03	41	13.44	20	6.56
Age average		52.12 ± 10.13		23.15 ± 4.24		38.51 ± 9.23	
Education	Diploma<	15	11.6	-	-	-	-
	Diploma	61	47.3	-	-	-	-
	Bachelor≥	53	41.1	-	-	-	-
Previously published article	Yes	-	-	-	-	13	24.7
	No	-	-	-	-	41	75.3
Editing article experience	Yes	-	-	-	-	6	10.8
	No	-	-	-	-	48	89.2
Scientific Level	Professor	-	-	-	2	-	3.7
	Associate Professor	-	-	-	7	-	13
	Assistant professor	-	-	-	45	-	83.3
H Index	≤ 2	-	-	-	-	15	27.8
	> 2	-	-	-	-	39	72.2

This study showed that 18.6% of the patients were pictured from their faces. In the opinion of 16.28% of the patients, no medical images must not be published. 79.84% of patients were against taking recognizable photos for integration in medical journals. 32.56% of patients believed they must be aware of their image usage. Table 2.

**Table 2:** Patient’s answer to the questionnaire questions

Question	Yes		No		No comment	
	No	%	No	%	No	%
Have you ever been photography from your mouth or face in the clinic or college?	24	18.60	105	81.40	0	0.00
Do you think that using identifiable images for all medical purposes is acceptable?	98	75.97	29	22.48	2	1.55
Do you think that no medical images should be published?	21	16.28	103	79.84	5	3.88
Do you agree with taking images to insert them in the case?	90	69.77	37	28.68	2	1.55
Do you agree with taking identifiable images to insert them into medical journals?	90	69.77	36	27.91	3	2.33
Do you agree with taking identifiable images to insert them into medical websites?	25	19.38	103	79.84	1	0.78
Do you agree with taking de-identifiable images to insert them into medical journals?	85	65.89	41	31.78	3	2.33
Do you think that using identifiable can hurt the individual?	85	65.89	42	32.56	2	1.55
Do you think that the patient must be aware of the usage of their images(face)?	41	31.78	83	64.34	5	3.88
Do you think that the patient must be aware of the usage of their images (oral and teeth)?	42	32.56	85	65.89	2	1.55

1. 74.07% of professors had used images of patience in teaching. The answers to the professors about: “do you agree with asking permission to integrate into the case? Do you agree to take de-identifiable photos for integration into medical websites? Do you agree to abide by de-identifiable photos for integration in medical journals?” were tabuleted in Table 3.
2. 1.64% of students had used images of patients in an article. 90.16% decided to take de-identifiable photos to integrate into medical websites. 12.30% of patients must be aware of the journal in which the medical images were published. Table 4.

**Table 3:** Teacher’s answer to the questionnaire questions

Question	Yes		No	
	No	%	No	%
Have you ever used patient images in teaching?	40	74.07	14	35.13
Have you ever taken a shot from a patient’s mouth or face in the clinic or college?	21	38.89	33	61.11
Do you think that using identifiable images for all medical purposes is acceptable?	54	100	0	0
Do you think that no medical images should be published?	2	3.70	52	96.30
Do you agree with taking images to insert them in the case?	54	100	0	0
Do you agree with taking identifiable images to insert them into medical journals?	6	10.2	48	89.8
Do you agree with taking identifiable images to insert them into medical websites?	6	10.2	48	89.8
Do you agree with taking de-identifiable images to insert them into medical journals?	54	100	0	0
Do you think it can hurt the individual?	54	100	0	0
Do you think that the patient must be aware of the usage of their images?	21	38.89	33	61.11

**Table 4:** Students’ answers to the questionnaire questions

Question	Yes		No		No comment	
	No	%	No	%	No	%
Have you ever used patient images in publishing an article?	2	1.64	120	98.36	0	0
Have you ever taken a shot from a patient’s mouth or face in the clinic or college?	2	1.64	120	98.36	0	0
Do you think that using identifiable images for all medical purposes is acceptable?	100	81.97	17	13.93	5	4.10
Do you think that no medical images should be published?	20	16.39	100	81.97	2	1.64
Do you agree with taking images to insert them in the case?	110	90.16	12	9.84	0	0
Do you agree with taking identifiable images to insert them into medical journals?	41	33.61	77	63.11	4	3.28
Do you agree with taking identifiable images to insert them into medical websites?	31	25.41	89	72.95	2	1.64
Do you agree with taking de-identifiable images to insert them into medical journals?	110	90.16	12	9.84	0	0
Do you think that using identifiable can hurt the individual?	110	90.16	12	9.84	0	0
Do you think that the patient must be aware of the usage of their images?	25	20.49	93	76.23	4	3.28
Do you think the patient must be aware of the specific journal in which their image is published?	15	12.30	106	86.89	1	0.82
Do you think it can hurt the individual?	32	26.23	88	72.13	2	1.64

As is shown in Table 5, the views of respondents among three groups were different. More than half of the patients (59.91%), 39.5% of students, and 31.48% of professors believed there was no need for permission, while only half of the students thought that the informed consent was needed for collecting patient clinical data.

**Table 5:** Participants’ viewpoints on the questions

Question 1	Patients		Students		Teachers		*p* value
	No	%	No	%	No	%	
Yes, the patients must always confirm	No	%	No	%	No	%	0.001*
Doctors can publish them without asking the patient’s permission	76	58.91	48	39.50	17	31.48	
I don’t know	21	16.28	10	8.20	2	3.70	
No comment	2	1.55	1	0.82	0	0	
Question 2							0.002*
The patient must see the whole article, although it is hard to comprehend in the written language	5	3.88	12	9.84	2	3.70	
The patient must see the article with the mother’s language translation	15	11.63	15	12.30	2	3.70	
The patient must see the image which is published in the article without the text	15	11.63	10	8.20	1	1.85	
There is no need for the patient to see the article or image before sending or being published in a journal	94	72.87	85	69.67	49	90.74	

*P* < 0.05 is significant

Question 1. Do you think asking permission to gather patients’ total data as a part of medical research, which will be published in a medical journal, is necessary?

Question 2. Do you think in case sending an article to a medical journal containing a patient’s images must:

Most of the patients and professors believed that it is unnecessary to show the pictures or publish papers to the patients. On the other hand, 12.3% of the students thought the patients must see the entire article, preferably with a translation to their native language.

Regarding patient images, a general process existed in all three respondent groups; in the opinion of the respondents with an increasing level of identification, strict consent forms are needed (Table 6). When the whole face is shown in an image, 64.34% of patients, 89.34% of students, and 83.33%of professors believed that written permission for publishing is needed. After controlling age and gender, professors have more desire to receive consent for using any image than patients. With a decreasing level of identification, doctors and students have more desire to receive approval than patients. Table 7.

**Table 6:** Participants’ views on necessity of consent for publishing patient’s clinical images with different levels of de-identification

Question		Patients		Students		Teachers	
		No	%	No	%	No	%
The patient’s verbal consent is enough	Radiographical image of the upper and lower jaw	110	85.27	20	16.4	54	100
	Image of the oral cavity	105	81.40	20	16.4	54	100
	Image of patient’s face with blurring eye area	19	14.7	51	41.80	2	3.70
	Full image of the face without de-identification	15	11.63	2	1.64	2	3.70
	Image of the hand	20	15.50	89	72.95	54	100
The doctor must take written consent	Radiographical image of the upper and lower jaw	10	7.7	1	0.82	0	0
	Image of the oral cavity	15	11.6	1	0.82	0	0
	Image of patient’s face with blurring eye area	10	7.75	15	12.30	41	75.93
	Full image of the face without de-identification	83	64.34	109	89.34	45	83.33
	Image of the hands	10	7.75	5	4.10	0	0
The doctor can publish them without asking the patient’s permission.	Radiographical image of the upper and lower jaw	9	7	101	82.8	0	0
	Image of the oral cavity	9	7	101	82.8	0	0
	Image of patient’s face with blurring eye area	100	77.52	56	45.9	11	20.4
	Full image of the face without de-identification	102	79.07	11	9	7	12.9
	Image of the hand	99	77	28	22.9	0	0

**Table 7:** Need of total consent against no need for aware approval for publishing a patient’s image with different levels of de-identification (the question about the appearance of the entire face is deleted due to lack of difference between responses)

	Image of the oral cavity		Radiographical image of the upper and lower jaw		Image of the hands		Photograph of patient’s face with blurring eye area	
	(95 CI) OR	*P*	(95 CI) OR	*P*	(95 CI) OR	*P*	(95 CI) OR	*P*
Age	1.000 (0.823–1.104)	0.213	1.016 (0.872–1.124)	0.467	1.034 (0.889–1.045)	0.321	1.021 (0.883–1.034)	0.07
Gender								
Men	Reference	-	Reference	-	Reference	-	Reference	-
women	1.134 (0.734–1.405)	0.325	1.431 (0.890–1.879)	0.145	1.671 (1.124–2.156)	0.011*	1.701 (1.28–2.432)	0.013*
Group								
Patients	Reference	-	Reference	-	Reference	-	Reference	
Students	1.546 (0.876–2.765)	0.084	2.542 (1.521–3.743)	0.002*	2.321 (1.310–3.652)	0.007*	2.121 (1.154–3.542)	0.01*
Teachers	2.134 (1.453–3.103)	0.001*	2.723 (1.543–3.879)	0.001*	2.654 (1.562–4.345)	0.001*	2.481 (1.641–3.425)	0.001*

*P < *0.05 is significant

As shown in Table 8, dentistry professors and students emphasized more than patients on reobtain approval for publishing necessary images. Finding revealed that the decrease in de-identified level leads to increasing the desire to receive consent with this fact that there is no significant difference between professors and patients. Besides, as the level of identification increases, the necessity of receiving written consent also increases.

**Table 8:** Need for oral or written consent against no need for aware approval for publishing a patient’s image with different levels of de-identification (the question about the image of the whole face is deleted due to lack of difference between responses)

	Image of the oral cavity		Radiographical image of the upper and lower jaw		Image of the hands		Photograph of patient’s face with blurring eye area	
	OR (95 CI)	*P*	OR (95 CI)	*P*	OR (95 CI)	*P*	OR (95 CI)	*P*
Age	1.041 (0.945–1.01)	0.741	1.009 (0.985–1.105)	0.254	1.102 (0.925–1.125)	0.145	0.985 (0.985–1.019)	0.542
Gender								
Men	Reference	-	Reference	-	Reference	-	Reference	-
women	1.124 (0.845–1.190)	0.84	1.245 (0.914–1.654)	0.123	1.542 (1.074–2.008)	0.024*	1.452 (0.785–2.412)	0.143
Group								
Patients	Reference	-	Reference	-	Reference	-	Reference	-
Students	1.876 (1.12–3.456)	*0.032	2.234 (1.234–4.456)	0.004*	2.789 (1.345–5.456)	0.001*	6.678 (1.976–23.345)	0.002*
Teachers	2.890 (1.546–4.786)	*0.001	4.567 (2.345–6.567)	0.001*	4.767 (2.456–7.654)	0.001*	11.234 (2.76-50.789)	0.001*

*P* < 0.05 is significant

## Discussion

This study investigates professors, students, and patients’ opinions about publishing patients’ images (Null hypothesis). This research showed that viewpoints on the necessity of asking permission to publish clinical manifestations differ between patients, students, and professors and specialist’s had a more positive attitude. This finding is in agree with Roguljić’s et al., Chassang and Villamañán et al. [26–28].

When it was asked about permission to publish a patient image from participants, more than half of the patients and almost one-third of professors and students thought that there is no need for the license about posting patients’ photos. Patients were more flexible in using clinical images with a high de-identification level, but they became most strict when the whole face is shown. However, 39.5% of students and 31.3% of professors believed that even for this kind of image, no consent is needed from the patient.

Professors and students had a most strict viewpoint about asking patients about confirmation for publishing clinical images, but 4% of them still think patients’ oral consent for publishing face full images is enough. When asked about observing an article in which the photos were published, patients and professors believed that there is no need to show printed images or reports to the patients. On the other hand, 12.3% of students thought patients must see the whole article with a rather mother language translation.

Receiving a consent letter is accepted as a general principle among medical staff. Still, there are disagreements about the way of receiving the letter of approval. the terms and cases of medical works which need to obtain one, and also the practice of giving information and explaining the benefits and damages of medical outcomes and the people who must be given the information to and also the size of the provided information to patient [28–30].

Moreover, sometimes submitted letters of authorization by patients or taken by medical staff can be without any legal value because of medical staff unawareness and failure to accept aware consent terms.

Roguljić’s et al. [27] showed that when medical information is published without medical images, only half of the patients and about 70% of students or professors knew that informed consent is needed. Conscious consent allows the patients to balance between potential benefits and damages of sharing information and controlling their data.

However, given aware letters of authorization is long and complicated, and there is a possibility that the patients do not understand them or do not read them completely [31, 32]. It is shown that even after giving aware consent about health treatment, the patients forget the benefits and risks after a short time period. So, giving enough additional information will be helpful to the patients.

Therefore, holding medical courses of ethics for students and professors emphasizes the importance of aware consent and helps the patients to read and understand aware letters of authorization. Studied cases in medical resources contain different information about patients, which even make patients’ identities recognizable. However, asking for informed consent from the patients before publishing any report is still in doubt [33, 34]. International medical editors of medical articles claimed that before writing any explanation of the patients in which complete de-identification is not possible, aware consent is needed [35].

In the present research, we used a questionnaire pattern, and there is the possibility that participants give socially desirable answers, such that answers to the first questions in the questionnaire affect given answers to further questions. However, patients may have different viewpoints about their images; also, professors/students make other decisions about the actual use of images. Also, because the sample of patients was self-chosen, they were interested to participate in the research and felt doubtful of their doctor. This matter can be the reason for the difference between the two studies.

Although doctors and patients increasingly use the internet for searching for medical information, it is not clear that they are aware of the fact that under the protection of published literature -CCBY – the reuse of medical images is not controllable. Also, journal policies are different in publishing patients’ appearances. For example, in eastern and southwest Europe, only in policies of 24% of medical journals, patient privacy and trust ability of medical information are considered into account [36–39].

Flexibility in giving consent to publishing medical images is related to less awareness of medical morals, general morals, and matters of protecting patients’ privacy in professional comments. Also, it is shown that, patients have high trust ability in health specialists, generally. Also, they believe that they are competent in making important medical data management decisions and are obedient in doctor-patient relationships [32, 33]. Although doctor-patient relation is a shared decision model in some counties, the dominant connection is from the doctor, which can affect the patient’s decisions [29].

The result of this study can be affected by particular circumstances. For example, there is a possibility that patients’ answers are influenced by this fact that they do not make fundamental decisions about their images. When the patients make accurate decisions, their points of view differ depending on the circumstances of the stage of the disease. Also, most of the patients and doctors thought that to understand the primary purpose of the images used in the article, there was no need to give the published paper to the patients. This result is in agree with the findings by Roguljić’s et al. [27].

Different elements, especially educational levels, can affect patients’ decisions to publish their medical images. The patients in this research were adults who had complete ability to make health decisions and post pictures, similar to other studies [40]. Before beginning the investigation, the questionnaire was tested by different patients to ensure that the content is written in a language in which reading and understanding are easy. Therefore, patients’ answers are not influenced by the language of the complexity of the subject.

This study showed that with an increased level of identification possibility, patients showed little desire to permit publishing. However, only one-third of patients had this idea that a written letter of consent is needed for publishing a full-face image without de-identification. Professors and dentistry students had a most strict opinion about the necessity of patients’ consent for publishing medical images than the patients; because the students were more trained than the patients. The professors had the more strict viewpoint, probably because of more medical experience and learning medical ethics, and therefore had more desire to hold ethical matters than the student, which complies with Roguljić et al. [27] and Lie et al. research [41].

Studies show that although students studying medicine and dentistry have similar ethical education during their education, dentistry doctors and students have more strict viewpoints about the necessity of consent for publishing medical images than medical doctors and students. A possible explanation for this difference is that dental specialists focus more on the face and mouth than other students and doctors, resulting in more awareness of moral de-identification matters. Very little research is done about patients’ viewpoints on publishing medical images.

In Lou et al. [37], British patients’ viewpoints were asked about using their medical images for medical purposes or publishing articles. Participants were asked about “identifiable” and “unidentifiable face images without any additional details of the field of medical images level of responsibility. Also, they were asked express their satisfaction with using the images for medical purposes of other patients, education, and article publication as a score. The patients preferred unidentifiable medical images rather than identifiable images, and mostly they answered that consent is needed for any use of the images. Adimo et al. [42] with a similar method to Lou et al. [37], analyzed acceptance of comprehension of medical images among Nigerian patients. Nigerian patients also preferred unidentifiable images and mainly expressed regardless of the purpose of use; their consent is needed.

In many cases, no guidance was given about acquiring patients’ consent for publishing their images. According to present moral guidance, patients’ data and information (unlike total data like clinical tests) must be only published in the event of existence [2, 4, 43–45].

Regarding the publishing articles with medical images, publishers and editors have tried to develop ways to decrease the possibility of being recognized while maintaining patient privacy. These efforts are complicated because of containing the facial image. The most common way is printing a black line on the eyes, or more common, blacking or Pixelizing the eyes area. Although this method is still being used, it generally is not considered acceptable because it does not protect patient privacy. Some articles suggest that anti-diagnostic items are no longer necessary when aware consent is suitably acquired [4].

Nevertheless, the current study and Roguljić et al. [27] research showed that medical and dental professors and students don’t have enough information on the importance of acquiring written consent in all previous cases before publishing. Publishing patients’ single images or their data must be done only in case of completion of the consent procedure. It usually means the patients delivered the written consent for publishing. This consent must be different from any other consent about treatment for participation in research. Preferably, the patients must be allowed to read the copy and receive information about the subject of pictures (and their possible reuse) before verifying. Although, there may be technical problems in providing the document to patients; for example, if they do not understand the language of the context (like English), in the case of children or disabled, they have problems following the technical language of articles. For these problems, they need specific guidance on this matter [4].

Assessing the opinions of experts and patients regarding the publication of patients’ facial images is important for improving the delivered health care services. This study showed that more strict forms are needed by increasing identity recognizability. The professors are more eager than the patients to receive patients’ permission for any kind of image. By reducing the level of identification, doctors and students are more eager than patients to receive approvals. Therefore, it seems that more studies are needed in this field and the level of awareness of patients should be raised. Future research must also investigate patients’ desire to post their medical photos compared to their decisions about someone else’s image. The originality of this article is because it is the first study in the Middle East. Both patients, professors and students have been included in this study and their opinions have been weighed together. The results of this study can be helpful regarding the importance of obtaining the consent of patients regarding the publication of their images and raising the level of awareness of professors and students regarding the publication of patient images.

## Conclusion

More than half of the patients and one-third of professors and students believe that no consent is needed for publishing patients’ images. Also, most patients and professors have this idea that there is no need to show the published article or pictures to the patients. A stricter consent form is needed in participants’ viewpoints with increasing identity recognition. After controlling gender and age, professors desire to acquire consent for using any image rather than the patients.

## Limitation

Non-cooperation of some participants.

Focusing on patients who have referred for dental work.

The use of a questionnaire that can be used in subsequent works of qualitative research.

The lack of understanding of a number of patients about the concept of publishing their pictures in journals.

## Data Availability

The datasets used and/or analyzed during the current study available from the corresponding author on reasonable request.
